# Society Notice

**Published:** 1864-06

**Authors:** Wm. O. Kulp

**Affiliations:** Cor. Secretary


### SOCIETY NOTICE.

The IOWA STATE DENTAL SOCIETY holds its next meeting at the city of Davenport, beginning Tuesday, the 9th day of August, 1864, at 8 o'clock P. M.

I take this occasion to urge upon you the great importance of an energetic and constant effort on the part of each and every member of the profession in the State of Iowa, to elevate its standard to that of other States, and by so doing we can the more satisfactorily convince the public of our usefulness and necessity as a profession. Such a result every true and honorable dentist most certainly looks forward to.

If you are not a member, we wish you to come and join us at once, and request any of your acquaintances whom we may not be able to address, to do the same.

Business of importance will be transacted at the approaching session, and reports from members who have visited the National Dental Societies will be presented. We also expect addresses from prominent Dentists from abroad. Your *personal attendance* is most respectfully solicited.

Arrangements will be made to board those attending this meeting at reduced rates.

SUBJECTS FOR DISCUSSION.

1. Filling Teeth.
2. Sensitive Dentine.
3. Alveolar Abscess—Causes and Treatment.
4. Deciduous Teeth and Irregularities.
5. Extracting Teeth.
6. Mechanical Dentistry.
7. Professional Etiquette.
8. Miscellaneous Subjects.

DR. MYERS,<br>
" COULSON, } *Committee.*<br>
" HARRIS,

WM. O. KULP, *Cor. Secretary.*

